# The increased gray matter volumes of precentral gyri in Parkinson's disease patients with diphasic dyskinesia

**DOI:** 10.18632/aging.102412

**Published:** 2019-11-07

**Authors:** Yan Zhi, Min Wang, Yong-Sheng Yuan, Yu-Ting Shen, Ke-Wei Ma, Cai-Ting Gan, Qian-Qian Si, Li-Na Wang, Sheng-Wu Cao, Ke-Zhong Zhang

**Affiliations:** 1 Department of Neurology, The First Affiliated Hospital of Nanjing Medical University, Nanjing 210029, PR China; 2 Department of Radiology, The First Affiliated Hospital of Nanjing Medical University, Nanjing 210029, PR China; 3 Department of Neurosurgery, The First Affiliated Hospital of Nanjing Medical University, Nanjing 210029, PR China

**Keywords:** Parkinson’s disease, diphasic dyskinesia, precentral gyrus, voxel-based morphometry, cortical thickness

## Abstract

Abnormal dopaminergic modulation of the cortico-basal ganglia motor loops results in the emergence of levodopa-induced dyskinesia (LID). We focused on alterations in the gray matter (GM) volume and the cortical thickness of the brain, especially in cortico-basal ganglia motor loops, in Parkinson’s disease (PD) with diphasic dyskinesia. 48 PD patients with diphasic dyskinesia, 60 PD patients without dyskinesia and 48 healthy controls (HC) were included. Voxel-based morphometry (VBM) was applied to get GM images from MRI brain images. FreeSurfer was used to get cortical thickness. Distinct analyses of covariance (ANCOVA) and linear contrasts were performed for early- and late-onset PD groups. The severity of diphasic dyskinesia was evaluated by the Unified Dyskinesia Rating Scale (UDysRS). Finally, the correlations between mean volumes of clusters showing differences and the UDysRS scores were performed by Pearson’s correlation. The GM volumes of precentral gyri were increased in PD patients with diphasic dyskinesia when compared with those without dyskinesia, which were positively correlated with UDysRS scores in PD patients with diphasic dyskinesia. However, there was no significant difference in cortical thickness among groups. The increased precentral gyri GM volumes might be associated with the pathogenesis and the severity of diphasic dyskinesia.

## INTRODUCTION

Parkinson’s disease (PD) is the second most common neurodegenerative disease after Alzheimer’s disease (AD) in the elderly people. For over 50 years, levodopa has been the best drug to treat the motor symptoms of PD [1]. However, levodopa-related motor complications including levodopa-induced dyskinesia (LID) and on-off phenomenon limits its usefulness [2]. What’s worse, motor complications not only have a bad effect on the quality of life but also increase treatment cost for PD patients [3]. LID is broadly divided into peak-dose dyskinesia, wearing-off or off-period dyskinesia, and diphasic dyskinesia according to their clinical movement patterns and the temporal correlation between the occurrence of dyskinesia and the levodopa pharmacokinetic changes [4, 5]. Diphasic dyskinesia, initially termed “dyskinesia-improvement-dyskinesia” or “beginning-/end-of-dose dyskinesias”, appear typically at the onset and end of levodopa antiparkinsonian action, coinciding with ascending and descending levodopa levels in the plasma [6]. Among three main categories of LID, diphasic dyskinesia is the most difficult to treat [7]. The reported prevalence of diphasic dyskinesia showed a wide range (from 3% to 20%) in PD patients showing motor fluctuations because of different study methods, selection of patients and patient populations [6]. Up to now, few studies focus on the pathogenesis of diphasic dyskinesia and it is still poorly understood.

Cerasa et al. have carried on several studies to explore alterations in the structure of brain in PD patients with LID [8–10]. They reported that dyskinetic group showed higher gray matter (GM) volume in the bilateral inferior frontal gyrus than nondyskinetic group [8, 9]. Moreover, they found a pronounced increase of thickness in the right inferior frontal sulcus in dyskinetic group when compared with nondyskinetic group [10]. Furthermore, they thought that age at onset influenced neurodegenerative processes underlying PD with LID [9]. However, dyskinetic group enrolled in their studies was limited to PD patients with peak-dose dyskinesia. As LID includes three categories, their results cannot be suitable for all LID. There are major discrepancies between peak-dose dyskinesia and diphasic dyskinesia. On the one hand, the pattern of peak-dose dyskinesia is the “improvement - dystonia - improvement” and it occurs around the time of peak plasma levels of medication [11]. The pattern of diphasic dyskinesia is the “dystonia - improvement - dystonia” and it occurs when the effects of levodopa are wearing on and off [12]. On the other hand, the performance of peak-dose dyskinesia is usually choreoathetoid, but can also be ballistic or dystonic [4]. Diphasic dyskinesia occurs more often in the lower limbs than the upper limbs, and it often consists of repetitive alternating movements in a stereotyped manner [6, 13]. Abnormal dopaminergic modulation of the cortico-basal ganglia motor loops is widely acknowledged to result in the emergence of LID [14]. Based on completely different clinical features between peak-dose dyskinesia and diphasic dyskinesia, we hypothesized that there were characteristic features in the structure of the cortico-basal ganglia motor loops, especially where associated with somato-motor function, which might be different from peak-dose dyskinesia.

In this study, we were the first to conducted a cross-sectional study to investigate alterations in the GM volume and the cortical thickness of the brain in PD patients with diphasic dyskinesia. Moreover, we conducted this study in early-onset PD patients (less than 50 ages) and late-onset PD patients (more than 50 ages) separately in order to take age at onset into consideration.

## RESULTS

### Demographic and neuropsychological characteristics

Demographic and neuropsychological characteristics of all participants were shown in Table 1. There were no significant differences in gender, age, education, MMSE scores and HAMD scores among groups. Moreover, no significant differences in disease duration, H&Y stage, LEDD and UPDRS-β scores were detected between PD patients with diphasic dyskinesia and PD patients without dyskinesia.

**Table 1:** Demographic data of participant.

**Items**	**EO PD with diphasic dyskinetic (n=17)**	**EO PD patients without dyskinesia (n=16)**	**HC (n=12)**	**p**
Gender (male/female)	9/8	10/6	9/3	0.48^a^
Age (years)	51.18±9.54	51.75±4.67	56.75±2.30	0.07^b^
Education (years)	10.71±3.51	10.81±2.90	12.58±3.61	0.28^b^
Disease duration (years)	10.41±5.15	8.37±4.02	NA	0.22^c^
Age at onset (years)	40.76±9.05	43.38±6.249	NA	0.35^c^
H&Y stage	2.53±0.41	2.50±0.37	NA	0.83^c^
LEDD (mg/day)	650.00±201.75	693.13±209.78	NA	0.55^c^
UPDRS-III	37.88±8.87	35.94±11.53	NA	0.59^c^
MMSE	28.94±0.90	28.63±1.50	28.75±1.22	0.76^b^
HAMD	4.24±2.73	5.13±3.20	4.42±2.64	0.66^b^
UDysRS	54.65±19.67	NA	NA	NA
**Items**	**LO PD with diphasic dyskinetic (n=31)**	**LO PD patients without dyskinesia (n=44)**	**HC (n=36)**	**p**
Gender (male/female)	16/15	24/20	23/13	0.56^a^
Age (years)	66.65±7.01	66.20±6.05	65.61±3.80	0.76^b^
Education (years)	10.42±4.22	11.02±3.55	11.19±3.29	0.67^b^
Disease duration (years)	8.45±2.89	8.43±2.31	NA	0.97^c^
Age at onset (years)	58.19±6.77	57.77±6.16	NA	0.78^c^
H&Y stage	2.58±0.53	2.50±0.61	NA	0.56^c^
LEDD (mg/day)	745.08±272.62	659.49±191.82	NA	0.11^c^
UPDRS-III	36.55±8.921	35.93±10.08	NA	0.79^c^
MMSE	28.16±1.42	28.30±1.76	28.69±1.43	0.34^b^
HAMD	4.61±2.83	4.75±3.85	4.08±2.76	0.64^b^
UDysRS	40.71±20.21	NA	NA	NA

Abbreviations: EO, Early-Onset; LO, Late-Onset; PD, Parkinson’s disease; HC, Healthy control; H&Y stage, Hoehn and Yahr stage; LEDD, levodopa-equivalent daily dose; UPDRS, Unified Parkinson’s disease rating scale; MMSE, Mini-Mental State Examination; HAMD, Hamilton Depression Scale; UDysRS, Unified Dyskinesia Rating Scale.

Values except gender were expressed as mean±S.D.

^a^Chi-square tests.

^b^Two-way analysis of variance (ANOVA).

^c^independent-sample t tests

*p<0.05

### VBM findings

**Figure 1:**
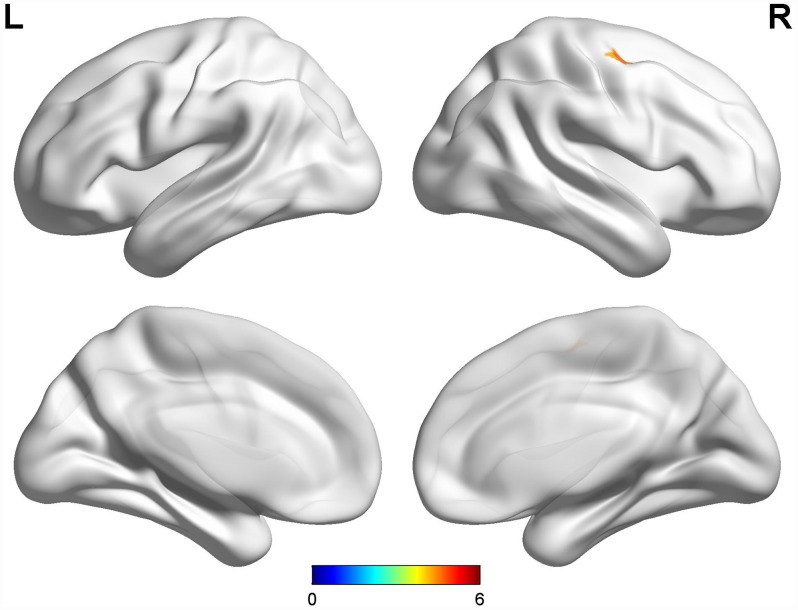
**Comparison between early-onset PD patients with diphasic dyskinesia and early-onset PD patients without dyskinesia.** Compared with early-onset PD patients without dyskinesia, early-onset PD patients with diphasic dyskinesia showed higher GM volumes in right precentral gyrus (Cluster size: 162 mm^3^). Correction for multiple comparisons (family-wise error rate (FWE), P < 0.05) was used to threshold the analysis. Abbreviations: PD, Parkinson’s disease; GM, gray matter.

**Figure 2:**
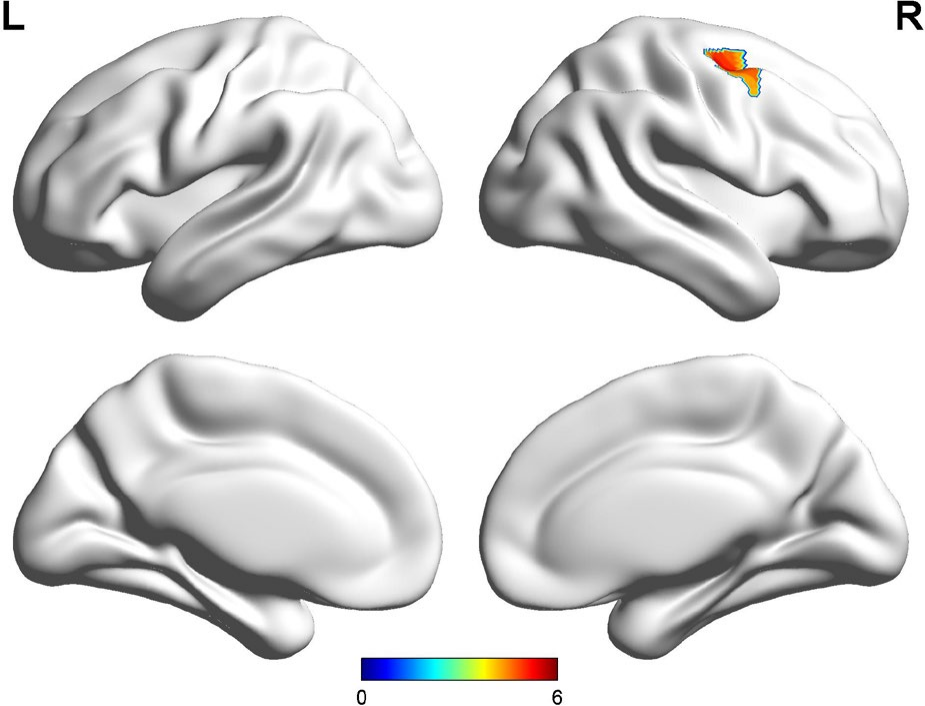
**Comparison between early-onset PD patients with diphasic dyskinesia and HCs.** Compared with HCs, early-onset PD patients with diphasic dyskinesia showed higher GM volumes in right precentral gyrus (Cluster size: 891 mm^3^). Correction for multiple comparisons (family-wise error rate (FWE), P < 0.05) was used to threshold the analysis. Abbreviations: PD, Parkinson’s disease; HC, Healthy control; GM, gray matter.

Compared with early-onset PD patients without dyskinesia, early-onset PD patients with diphasic dyskinesia showed higher GM volumes in right precentral gyrus (MNI local maxima: x = 33, y = −9, z = 54, F = 4.66, Cluster size: 162 mm^3^, P_FWE-corr_ < 0.02) (Figure 1). Moreover, early-onset PD patients with diphasic dyskinesia showed higher GM volumes in right precentral gyrus (MNI local maxima: x = 33, y = −6, z = 54, F = 5.16, Cluster size: 891 mm^3^, P_FWE-corr_ < 0.01) (Figure 2) than healthy controls (HCs). There was no significant GM difference between early-onset PD patients without dyskinesia and HCs at a threshold of FWE-corrected P < 0.05.

**Figure 3:**
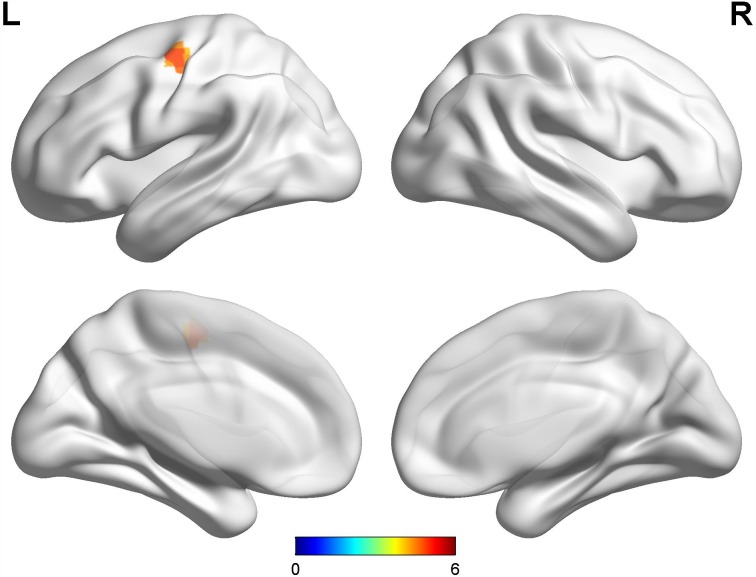
**Comparison between late-onset PD patients with diphasic dyskinesia and late-onset PD patients without dyskinesia.** Compared with late-onset PD patients without dyskinesia, late-onset PD patients with diphasic dyskinesia showed higher GM volumes in left precentral gyrus (Cluster size: 189 mm^3^). Correction for multiple comparisons (family-wise error rate (FWE), P < 0.05) was used to threshold the analysis. Abbreviations: PD, Parkinson’s disease; GM, gray matter.

Compared with late-onset PD patients without dyskinesia, late-onset PD patients with diphasic dyskinesia showed higher GM volumes in left precentral gyrus (MNI local maxima: x = -39, y = -12, z = 54, F = 4.91, Cluster size: 189 mm3, P_FWE-corr_ < 0.01) (Figure 3). There was no significant GM difference between HCs and late-onset PD patients with diphasic dyskinesia or late-onset PD patients without dyskinesia at a threshold of FWE-corrected P < 0.05.

### Cortical thickness findings

The data of cortical thickness was provided in Supplementary Table 1. There was no significant difference in cortical thickness among groups at a threshold of Bonferroni-corrected P < 0.05.

### Correlation analysis

**Figure 4:**
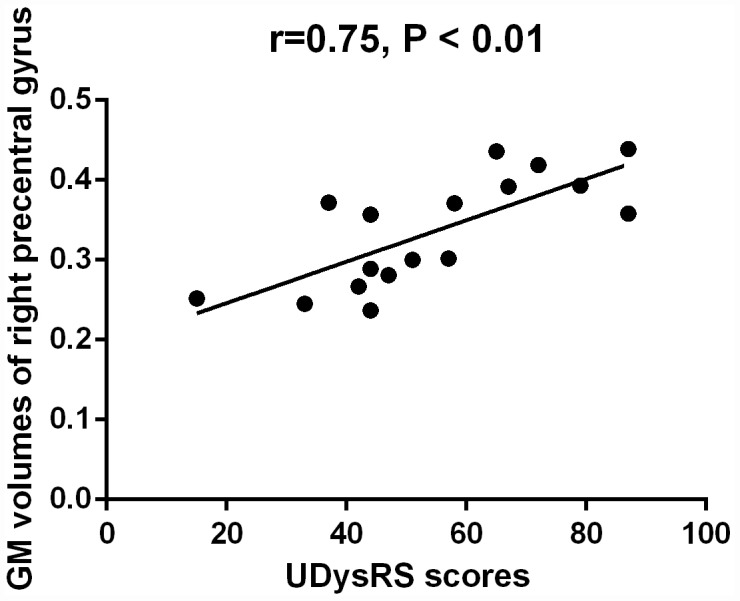
**Correlation between the mean GM volumes of the cluster showing difference and UDysRS scores in early-onset PD patients with diphasic dyskinesia.** The mean GM volumes of right precentral gyrus was positively correlated with UDysRS scores (r=0.75, P < 0.01).

**Figure 5:**
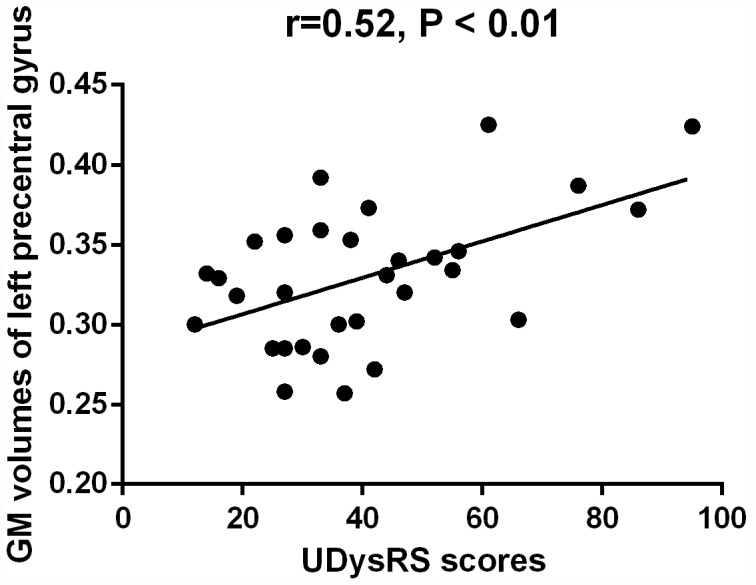
**Correlation between the mean GM volumes of the cluster showing difference and UDysRS scores in late-onset PD patients with diphasic dyskinesia.** The mean GM volumes of left precentral gyrus was positively correlated with UDysRS scores (r=0.52, P < 0.01).

The mean GM volumes of the cluster which showed differences between early-onset PD patients with diphasic dyskinesia and early-onset PD patients without dyskinesia in right precentral gyrus was positively correlated with UDysRS scores in early-onset PD patients with diphasic dyskinesia (r = 0.75, P < 0.01) (Figure 4). Moreover, the mean GM volumes of the cluster which showed differences between late-onset PD patients with diphasic dyskinesia and late-onset PD patients without dyskinesia in left precentral gyrus was positively correlated with UDysRS scores in late-onset PD patients with diphasic dyskinesia (r = 0.52, P < 0.01) (Figure 5).

## DISCUSSION

Our analysis showed that the GM volumes in right precentral gyrus were higher in early-onset PD patients with diphasic dyskinesia when compared with early-onset PD patients without dyskinesia. The GM volumes in left precentral gyrus were higher in late-onset PD patients with diphasic dyskinesia when compared with late-onset PD patients without dyskinesia. The difference might be attributed to unilateral limb onset in PD and the sample size. What’s more mean GM volumes of clusters in precentral gyri which showed differences were positively correlated with UDysRS scores, indicating that the increased GM volumes of precentral gyri might be associated with the severity and the pathogenesis of diphasic dyskinesia. However, there was no significant difference in cortical thickness among groups. The main cause might be that the increased GM volumes were the portion of precentral gyri and the cluster size was small. The cortical thickness related data got from FreeSurfer were mean cortical thickness of each brain region. The mean cortical thickness of the whole precentral gyri showed no difference among groups, indicating changes of precentral gyri were partial. Previous studies about peak-dose dyskinesia reported that GM volumes and thickness in the inferior frontal gyrus was higher in dyskinetic group when compared with nondyskinetic group [8–10]. We found that alterations in the structure of the brain in diphasic dyskinesia were different from peak-dose dyskinesia, which supported the hypothesis that alterations in the GM volume of the cortico-basal ganglia motor loops might have characteristic features which is different from peak-dose dyskinesia, indicating that peak-dose dyskinesia and diphasic dyskinesia had different pathogenesis.

Although, the pathogenesis of LID is not well understood, it is widely acknowledged that abnormal dopaminergic modulation of the cortico-basal ganglia motor loops results in the emergence of LID [14]. Loss of nigral dopaminergic neurons is the feature of PD. Progressive degeneration of presynaptic nigral neurons leads to the large dopamine fluctuation in the brain [15]. It leads to abnormalities in the connectivity between the motor cortex and the striatum and establishes a functional disturbance in basal ganglia, which is associated with the generation of involuntary abnormal movements [5]. The role of dopamine in the striatum is to alter the response of medium spiny neurons (MSNs) in both the direct and indirect pathways to excitatory input from the corticostriatal pathway [16]. In the dyskinetic state, uncontrolled and excessive dopaminergic stimulation over the direct pathway (striatonigral) MSNs increases thalamocortical feedback [16, 17], which leads to thalamo-cortical pathway disinhibition and frontal motor area overactivation [18]. Then, the overactive frontal motor area produces exaggerated motor function. The precentral gyrus is the site of the primary motor cortex (Brodmann area 4). The different body parts in the primary motor cortex has a precise somatotopic representation. The clusters which showed increased GM volumes of precentral gyri in our study were mainly located in the upper of precentral gyrus. Hence, the increased GM volumes of precentral gyri might be associated with repetitive alternating movements in the lower limbs in PD patients with diphasic dyskinesia.

However, we couldn’t judge whether the increased GM volumes of precentral gyri were the consequence or the cause of diphasic dyskinesia. Previous studies have proved that impaired brain plasticity results in the development of dyskinesias during the long-term dopaminergic therapy in PD [19]. PD patients with LIDs show absent or poor plastic responses in the primary motor cortex, which cannot be compensated by chronic dopaminergic replacement therapy [19–21]. The normal bidirectional plasticity in both direct and indirect pathways was replaced by unidirectional plasticity (Long-Term Potentiation-like in the direct pathway and Long-Term Depression-like in the indirect pathway) in the rodent PD model with dyskinesia in the presence of levodopa [22]. We speculate that the GM volumes of increased precentral gyri might be the consequence of impaired brain plasticity during the long-term dopaminergic therapy. Another hypothesis is that the increased GM volumes of precentral gyri were normal variation from birth, and PD patients with increased GM volumes of precentral gyri were easier to appear impaired brain plasticity and develop diphasic dyskinesia. It’s hard to judge which hypothesis is right based on the current study, so further studies are necessary to be carried on to confirm related mechanisms.

It is worth noting that the development of diphasic dyskinesia could be influenced by several clinical variables including levodopa dose, age, gender, duration of disease, clinical subtype, disease progression and disease severity [4]. Therefore, we chose closely matched diphasic dyskinetic group and nondyskinetic group to minimize the possible influence of these clinical variables on cerebral structure. Moreover, gender, age and education were entered as covariate of no interest in our analysis to ensure the reliability of our study. Further correlation analysis showed that the increased GM volumes in precentral gyri were only associated with UDysRS scores, but not associated with disease duration, H&Y stage, LEDD and UPDRS-III scores, indicating that the increased GM volumes in precentral gyri were specific in PD patients with diphasic dyskinesia.

We must take several limitations in the present study into consideration. Firstly, this study was confined to investigate alterations in the GM volume and the cortical thickness of the brain, and the mechanism of diphasic dyskinesia should be further explored by other methods such as functional magnetic resonance imaging (fMRI) and diffusion tensor imaging (DTI). Secondly, we used a highly stringent statistical threshold (FWE < 0.05), so our study might be underpowered to detect some minor alterations in the GM volume of the brain in PD patients with diphasic dyskinesia. Thirdly, this is a cross-sectional investigation, and a prospective study would be better to observe dynamically cerebral change in the structure. Fourthly, the sample size of participants was relatively small. Therefore, results of our study need to be reproduced and validated in the further.

In conclusion, we present firstly the cerebral structural abnormalities in PD patients with diphasic dyskinesia. The GM volumes of precentral gyri were increased in PD patients with diphasic dyskinesia, which might be associated with the pathogenesis and the severity of diphasic dyskinesia. The structural characteristics of diphasic dyskinesia is different from peak-dose dyskinesia. The GM volumes of precentral gyri would be considered as biomarkers for diphasic dyskinesia in PD.

## MATERIALS AND METHODS

### Study participants

From June 2015 and September 2018, 114 well-characterized idiopathic PD patients who had received levodopa treatment more than six months were consecutively recruited from the First Affiliated Hospital of Nanjing Medical University according to the UK Parkinson’s Disease Society Brain Bank Research criteria [23]. Exclusion criteria were: (1) other forms of parkinsonism such as atypical parkinsonism and secondary parkinsonism, (2) a family history of PD, (3) structural abnormalities in the brain which might affect the gray matter, (4) head movement artifacts during the MRI Session, (5) severe cognitive impairment (mini-mental state exam (MMSE) score < 24) [24] or depression (Hamilton Depression Scale (HAMD) score > 17) [25]. As a result, six PD patients were excluded from our study (three PD patients because of head movement artifacts during the MRI Session, two patients because of severe cognitive impairment, one PD patient because of depression). The presence or absence of diphasic dyskinesia was judged by two experienced neurologists, Kezhong Zhang and Yongsheng Yuan, depending on involuntary movements appearance in the time window after an acute levodopa test on the occasion of the last visit at least one week before MRI examination, along with relevant clinical symptoms previously. Among 108 PD patients, 48 patients suffered from diphasic dyskinesia, and 60 patients were nondyskinetic. According to previous published criteria, each PD group was further divided into early-onset group (age < 50) and late-onset group (age > 50) [26]. Thus, PD patients were divided into four groups: early-onset PD with diphasic dyskinesia (n=17); late-onset PD with diphasic dyskinesia (n=31); early-onset PD without dyskinesia a (n=17) and late-onset PD without dyskinesia (n=43). Moreover, 48 healthy individuals without neurological diseases, a family history of PD or cognitive impairment were enrolled as controls. All HCs were matched for age, sex, and education with PD patients well. All participants gave us written informed consent before participating in the experiment and the study was approved by the ethics committee of the First Affiliated Hospital of Nanjing Medical University.

### Clinical assessment

We used the Hoehn and Yahr (H&Y) staging and the third part of the Unified Parkinson’s Disease Rating Scale (UPDRSIII) [27] to evaluate the motor severity of PD patients. Moreover, the Unified Dyskinesia Rating Scale (UDysRS) [28] was used to evaluated the severity of diphasic dyskinesia in diphasic dyskinetic PD group. The UDysRS is a comprehensive rating tool of dyskinesia in Parkinson’s disease [28]. It has been proved to have acceptable internal consistency, inter- and intrarater reliability, and temporal stability [28, 29]. Furthermore, the presence of depression was detected using the Hamilton Depression Scale (HAMD) [26]. Cognitive functioning of all participants were evaluated by the mini–mental state examination (MMSE) [25]. We calculated levodopa equivalent daily dose (LEDD) according to established methods [30].

### Image acquisition

All participants were fasting in the morning and to PD patients at least 12 hours withdrawal of levodopa treatment. Experienced doctors from the radiology department using a 3.0 Tesla Siemens MAGNETOM Verio whole-body MRI system (Siemens Medical Solutions, Germany) which was equipped with eight-channel and phase-array head coils to scan all participants. Ear-plugs were used because of the big noise made by the scanner. Moreover, tight foam padding was used to minimize head motion. Three-dimensional T1-weighted anatomical images were obtained using a volumetric 3D magnetization-prepared rapid gradient-echo (MP-RAGE) sequence (repetition time (TR) = 1900 ms, echo time (TE) = 2.95 ms, flip angle (FA) = 9°, slice thickness = 1 mm, slices = 160, field of view (FOV) = 230×230 mm^2^, matrix size = 256×256 and voxel size = 1×1×1 mm^3^).

### Voxel-based morphometry (VBM)

Structural data analysis and atrophy measurements were performed with optimized VBM [31]. Statistical Parametric Mapping (SPM8, http://www.fil.ion.ucl.ac.uk) software was used to process and examine MRI data preprocessing. Three PD patients with head motions exceeded 2.5 mm or 2.5° of translation or rotation in any direction throughout the course of the scan were excluded in our study VBM standard routines and default parameters implemented were applied in the VBM8 toolbox (http://www.neuro.uni-jena.de/vbm/). The DARTEL toolbox was incorporated with the VBM8 toolbox to obtain a high-dimensional normalization protocol. Firstly, images were bias-corrected, tissue-classified, and registered by a generative model of VBM preprocessing which was provided by unified segmentation. Secondly, the segmented GM images were normalized by the Montreal Neurological Institute (MNI) brain. After that, GM images were modulated by the Jacobian determinants derived from the spatial normalization. Finally, the modulated volumes were smoothed with a Gaussian kernel of 8 mm full width at half maximum.

Two doctors who had no knowledge of the participants statistically analyzed the GM volume maps using the general linear model based on Gaussian random field theory. The smoothed GM images were entered into a second-level analysis of covariance (ANCOVA) model to evaluate the main effect of group. In order to rule out the possibility that GM differences caused by specific factors, gender, age, education and total intracranial volume were entered as covariate of no interest in the ANCOVA analysis. Distinct ANCOVA analyses were performed for early- and late-onset PD groups. Linear contrasts were used to test for differences in GM volumes between the groups. Correction for multiple comparisons (family-wise error rate (FWE), P < 0.05) was used to threshold all analyses.

### Cortical thickness

Cortical thickness analysis was performed with the FreeSurfer software (http://surfer.nmr.mgh.harvard. edu/, version 6.0) using “recon-all” processing stream [32] which included: (1) motion correction and averaging of volumetric T1 weighted images, (2) removal of non-brain tissue including stripping of the skull, (3) automated Talairach transformation, (4) segmentation of the subcortical white matter and deep gray matter volumetric structures, (5) intensity normalization, (6) tessellation of the gray/white matter boundary, (7) automated topology correction, (8) surface deformation [33–35]. Two doctors who had no knowledge of the participants performed initial visual inspection of segmentations and minor manual corrections of regional segmentation. Then we extracted cortical thickness from all available cortical structures in both hemispheres. Comparison of cortical thickness from all available cortical structures was evaluated using ANOVA among groups. Gender, age and education were entered as covariate of no interest in the ANCOVA analysis. Distinct ANCOVA analyses were performed for early- and late-onset PD groups. Linear contrasts were used to test for differences in cortical thickness between the groups. Correction for multiple comparisons (Bonferroni, P < 0.05) was used to threshold analyses.

### Statistical analysis

SPSS 20.0 statistical analysis software (SPSS Inc. Chicago, IL, USA) was used to perform the analysis of demographic and neuropsychological data. Continuous variables and categorical variables were shown as mean±S.D and a percentage respectively. Chi-square test was used to compare gender among groups. Comparisons of age, education, MMSE scores and HAMD scores were evaluated using two-way analysis of variance (ANOVA). Moreover, independent-sample t tests were employed for the comparison of disease duration, H&Y stage, LEDD and UPDRS-Ⅲ scores between PD patients with diphasic dyskinesia and PD patients without dyskinesia. Furthermore, we extracted the mean GM volumes of clusters show differences between PD patients with diphasic dyskinesia and PD patients without dyskinesia. A imaging analysis program developed described in Yuan et al. [36] was used to perform mean GM volume measurements. Pearson correlation was employed to calculate correlations between mean GM volumes of clusters and UDysRS scores in PD patients with diphasic dyskinesia. Correlations between cortical thickness from cortical structures which showed difference and UDysRS scores in PD patients with diphasic dyskinesia were calculated by Pearson correlation, as also. Two tailed levels of significance (P < 0.05) was used.

## Supplementary Material

Supplementary Table 1
